# The Evolution of Gynæcology

**Published:** 1914-12

**Authors:** W. H. C. Newnham

**Affiliations:** Clinical Lecturer in Obstetrics in the University of Bristol


*"Scire est nescire, nisi id me*
*Scire alius sciret."*

DECEMBER, 1914.

### THE EVOLUTION OF GYNÆCOLOGY.

The Presidential Address, delivered on October 14th, 1914, at the opening of the Forty-first Session of the Bristol Medico-Chirurgical Society.

BY

W. H. C. NEWNHAM, M.A., M.B. Cantab.,

*Clinical Lecturer in Obstetrics in the University of Bristol.*

---

It is very difficult to express in words my appreciation of the honour conferred on me by this distinguished Society in selecting me as its President for the coming year. I know what a high record my predecessors have established, and in thanking you for the honour you have done me, I can only say I will try to do the best I possibly can for you.

Now one of my duties is to give you an opening address on the first day of the session, and I could wish it were possible for me to take a subject outside the profession; but unfortunately that is beyond my powers, and therefore I am driven to my own particular line, and I propose to give you quite a short paper on the evolution of gynæcology, an essay which I fear will be dry as dust to the majority of my unfortunate listeners.

In no department, I suppose, has the progress been greater in medicine than in that assigned to the diseases of women.

Fifty years ago there was a craze for inflammation and ulceration of the os uteri, when every woman in a household was supposed to be a sufferer, and was treated accordingly.

There was a very short period when clitoridectomy was strongly advocated, only to be abandoned. Every backache, pelvic discomfort, or general neurosis was attributed to a mechanical displacement, and had to be treated by uterine pessaries.

There also came a time when oöphorectomy was advised for restraining hemorrhage from bleeding fibroids, and also for various neurotic symptoms, until the dire results produced by these operations were found out.

It is always a pleasure to acknowledge the debt of gratitude we owe to Lord Lister, for without his scientific discoveries the success of modern pelvic surgery could never have taken place.

In 1845 H. J. Bennett wrote a book insisting, amongst other things, "that inflammation is the chief factor, and that displacements and ulcerations follow from it, that this inflammation is confined to the cervical canal, and does not affect the body of the uterus, and that the disease should be attacked by strong caustics."

Only sixty years ago Velpeau in France and Graily Hewitt in our own country stood out as champions of the theory that all uterine trouble was caused by some malposition of the organ, and so every gynæcologist felt bound to invent some new form of cradle or pessary, or to modify some existing form, so that, to quote the words of Clifford Allbutt, "the uterus could justly proclaim that it was always being impaled on a stem or perched on a twig," but there was no monotony about the twig.

While the current of gynæcology, as it has flowed down to us in an ever-widening stream from the past, is traceable largely to the Greeks, there is also evidence that it did not have its origin here, for the Greeks themselves wrote of the skill and learning of the physicians of Egypt.

The destruction of the Alexandrian Library has left the writings of Hippocrates, about 450 B.C., the oldest record containing anything like a systematic consideration of the diseases of women. He advised the use of aromatic fumigations in amenorrhea. May this be compared to the more modern suggestion of steaming the uterus? The woman who did not conceive was wrapped in blankets and fumigated from beneath. If the scent passed through her nostrils and her mouth, then it was known that she was not unfruitful. He also maintained that movement of the womb towards the head caused pain under the eyes and nose, with abundant frothy saliva; if it moved towards the hypochondrium it caused vomiting; if towards the liver, loss of speech and a livid skin. His knowledge of the sympathy between the uterus and mammæ is apparent, as he advises a large cupping instrument to the breast for uterine hemorrhage. The prolapsed uterus should be well washed with astringent lotions, restored to its place, and the woman placed on her back with the legs crossed and tied together. His description of cancer of the uterus is clear, and his prognosis is very gloomy.

Enough has been preserved of the writings of Galen and Celsus to show us that the speculum was not unknown, and that ulceration of the womb and leucorrhea in its several varieties had been recognised. In the excavations of Pompeii were found two specula which were probably in common use at the time, and had been buried for nearly eighteen hundred years.

In the old writings of Aëtius the description given of the methods for preventing the legitimate consequences of sexual congress and for inducing abortion proves that the nefarious practices by means of which the females of our day would accomplish the same result are not of modern origin.

That four volumes of Aëtius were devoted to diseases of women is a revelation to those who suppose that gynæcology is a development of later times.

The relief of stenosis by means of sponge tents makes one think that the modern discoverer of this treatment derived his idea from Aëtius, or else it is a wonderful coincidence. Except for a few references by various writers, the speculum was a lost instrument for a thousand years until its re-discovery (if such it really was) by Récamier in 1816.

In 1852 Marion Sims made surgery of the vagina and uterus a more exact science. His invention of the position of the body in which the speculum was used was what the printing press is to civilisation, the compass to the mariner, steam to navigation, the telescope to astronomy, and here it was grander because it was the work of one man. He used first of all a bent spoon handle, and finding this so efficacious directly the air rushed into the vagina, thus opening up a region that had never been properly inspected before. Directly after manufacturing the speculum he proceeded to operate on a very bad case of vesico-vaginal fistula, produced by himself with the use of forceps in a long-delayed confinement. The operation failed again and again, until at last he hit upon the device of using silver wire for his sutures, and this was crowned with success.

In 1830 Hodge first invented his celebrated pessary, the idea being evolved out of his brain one night while looking into the fire and noticing the supporting curve of a steel hook for supporting the shovel and tongs.

In 1876 at the American Gynæcological Society it was laid down as follows :—

1. Gonorrhœa persists for life in the male notwithstanding its apparent cure.

2. Latent gonorrhæa may exist in the male.

3. Latent gonorrhœa in the male may easily infect a perfectly healthy person.

4. Ninety per cent. of sterile women are married to men who have suffered from gonorrhœa previously or during their married life.

In my own personal experience a man having a supposed cure of gonorrhœa six years previously to marriage, and having no connection at all during that six years, infected his wife on the first night of his marriage, and also again developed the disease himself. Both were examined before marriage and passed as perfectly healthy, and were known to me for some years.

The ravages of gonorrhœa in a woman are hardly recognised, and the ordinary treatment of it is contemptible. Yet the virus of this disease gives rise to a group of pathological symptoms which surpass in importance any other class of affections with which the gynæcologist is called upon to deal. If gonorrhœa stood alone among the diseases of women that had been neglected for generations by the surgeons the explanation might be difficult ; but it has only shared what was the common lot with abdominal and uterine surgery, until the latter was removed from neglected subjects by the work of the specialists.

A French writer on venereal disease states that it is possible to cure a gonorrhœa so effectually that a man may safely get married forty-eight hours after an injection of nitrate of silver. This is surgery fit only for satyrs, and yet the practical outcome of such advice and treatment is the ruin of the health of innocent women.

There can be no possible doubt that a large number of the apparently puerperal septic cases are of gonorrhœal origin. It is also quite possible for these symptoms not to appear for fully a month after the birth of the child, and unfortunately such cases have occurred in my own personal experience, where there cannot be the shadow of a doubt about the diagnosis.

The danger of gonorrhœa in the woman lies in this fact; the infection from the vulva, vagina or urethra may extend to the mucous membrane of the uterus, thence to the tubes, ovaries and peritoneum, and so may end in acute peritonitis from the escape of pus in the Fallopian tubes into the peritoneal cavity.

If you attach any value to statistics some are interesting reading. For example, out of one thousand men questioned eight hundred had at some time or other of their lives suffered from gonorrhœa, and at least one man put it down to making water in the night air. Out of one hundred women who married men who had at some time been afflicted with gonorrhœa only ten remained healthy as regards their sexual organs.

Gonorrhœal infection in women gives rise to a group of diseases which, by reason of their clinical interest and their social and moral consequences, surpass in importance every other class of affections which claim the attention of the gynæcologist, who, if he fails to closely examine into this subject, repulsive though it may be apart from its scientific interest, must constantly fail in his duty to his patients. Gonorrhœa is the most wide-spread of all the infectious diseases with the exception of measles, and the disease is one of the most common causes of sterility.

There can be no question that it is one of the most frequent causes of a declining birth-rate, for it cripples an endless number of men, and renders legions of women sterile. It is highly probable that over 50 per cent. of the inflammatory pelvic troubles met with in young married women are due to gonococcal infection by the husband, who is ignorant of the fact that he has not been cured before marriage of some early indiscretion.

It is a disgrace to our profession that so many men are told they are fit to marry after an attack of gonorrhœa without being properly examined. It is forgotten that the gonococcus may lie unsuspected in the ducts and glands that surround the urethra in the male.

Syphilis is a relatively harmless disease. It may cause discomfort, distress and even much pain, but does it ever kill a woman ? If it does, where syphilis kills its tens gonorrhœa kills its thousands, and it would take the sufferings of a hundred cases of syphilis to make up for the long, weary years of agony of one case of gonorrhœal pyo-salpinx.

It is possible for there to be a complete absence of vaginal inflammation, for the gonorrhœa may be communicated directly to the uterus itself, and so may arise a case of acute pyo-salpinx. Where it goes straight through the tubes to the peritoneal cavity we may never get the initial story of vaginal infection.

A typical history is such as this : A woman, aged 30, beginning her menstruation at 14 years of age, is perfectly well up to 22, when she marries. A few weeks or months afterwards she has an ill-defined illness, which keeps her in bed for two or three weeks. She is told it is an attack of inflammation of the bowels, and though this subsides and she gets about again, she is never really well, always more or less an invalid and sterile ! The ova are hindered from leaving the organ which is their source, and consequently the impregnation by the male element becomes an impossibility.

Lawson Tait recorded many cases in which healthy women within a few days of marriage get a gonorrhœal perimetritis. One instance he gives that is particularly interesting: "A man loses his first wife with gonorrhœal septicæmia after her first parturition in the first twelve months; he marries again several years later, infects his second wife within a week of marriage, and has a recurrence himself." A case like this is absolutely conclusive of the harm that may be done by a latent gonorrhœa, unless both man and woman are telling most abominable falsehoods, and there is no reason to suspect them, as the man admits gonorrhœa seven years previous to his first marriage, and the second wife was a perfectly healthy woman of thirty years of age.

It is most distressing to have to believe that men will inflict the sufferings of gonorrhœa upon their wives within a few days of their puerperal trials. But however much we may deplore it, it has to be stated as a fact upon which there can be no dispute that we have the most serious complications arising directly from this cause in the majority of instances, and cases have actually come under my own observation. When we get the confession of a husband having conferred a gonorrhœa upon his wife, we are at once taken out of the category of frivolous anecdotes into the realms of fact.

I am quite convinced that a large number of puerperal deaths, which without due consideration are put down to puerperal fever, are usually caused by gonorrhœa.

In one year at Queen Charlotte's Hospital there were five deaths from puerperal fever so-called, and four of these had pyo-salpinx. Was not this due to the same source?

A man tells you he does not attach much importance to his discharge, so he gives it to his wife.

The evolution of abdominal surgery has proceeded entirely from the necessities of the special diseases of women, and the combination is so complete that it is unlikely it will ever again be separated.

In 1809 the first ovariotomy was recorded; it was successful, and the patient lived on for thirty-two years. The history of successful ovariotomy dates from the publication of Spencer Wells's book in 1864, and from this time onwards step by step we have reached our present success. At a very early period he did not allow anyone present who had been in contact with a septic case, and he kept his ward for abdominal surgery quite separated from sloughing septic cases, and he gave up all *post-mortem* work.

I saw my first ovariotomy at the age of fifteen. The operator did not even wash his hands or take off his coat, the instruments were placed on a blanket thrown over the patient's feet, the operation was done in twenty minutes, the pedicle was clamped outside the abdomen, and the clamp left on under a water dressing. On the third day after the operation I saw the case in the *post-mortem* room, with a large amount of stinking pus in the pelvis, and the man who made the *post-mortem* had three cases of puerperal fever in his own private practice, all of which were fatal during the following week. My first six cases seen in London later on all died; and the first case I had anything to do with in Bristol, in spite of many precautions, such as spraying the operation room for twenty-four hours previously, keeping all students out of the room, was fatal from septic purulent peritonitis. Now one may truthfully say that the percentage of deaths after operation for ovarian disease is practically *nil*.

In 1759 and 1791 cases are recorded in America of successful operation for ectopic pregnancy, and during the sixteenth century several cases are mentioned, but in such a vague manner that their exact nature cannot be determined; in 1614 a *post-mortem* is recorded of a ruptured two-months' ectopic pregnancy, in which it is actually mentioned that the tube was much enlarged and ruptured; but it remained for Lawson Tait to really introduce the successful operation. In 1881 he was simply staggered at the suggestion of the general practitioner who called him in that he should open the abdomen, but two years later he successfully did the operation on another case. Ectopic pregnancy is an accident which may happen to any wife in the most useful period of her existence. It was first reported by Albucasis in the eleventh century, and yet until 1882 life was allowed to ebb away without a hand being raised to help the woman. Now the idea is to ligature the tube at once, and then remove the clots and debris at leisure. It may also be taken for granted that any intra-peritoneal hæmatocele is in all probability due to an ectopic gestation.

In the first case I saw personally the woman was enormously shocked and obviously suffering from some internal hemorrhage. On opening the abdomen some of the blood was let out, but no real search was made for the source of the trouble, and the wound was sewn up, the woman naturally dying the same night. On discussing this and other cases with the late Mr. Coe, he told me he had never in all his experience ever seen a case of ectopic pregnancy, and really tried to throw a damper on my youthful enthusiasm for operating, as he called it.

It now naturally falls to the lot of the gynæcologist to do very many hysterectomies or myomectomies for fibroid tumour, but until we were rendered safe by the wonderful discoveries of Lister and Pasteur, these operations were absolutely unsafe. At one time these tumours were left alone, with the hope (proved to be utterly fallacious) that they would disappear at the menopause ; or else they were removed by an operation in which the pedicle was fixed in the abdominal incision by the aid of the serre-nœud clamp, and in many cases suppuration took place at this spot. The one persistent and universal characteristic of all perfected surgical procedures is simplicity, so now the ordinary supra-vaginal or sub-total hysterectomy holds the field for most of these operations. It must, of course, only be undertaken by a man who has had the chance of gaining adequate experience and practice, for no operation can be more fatal than this in the hands of an amateur, and death almost invariably follows within a week when proper precautions are not taken during the operation. It is well to remember Keith's words : "It is unfortunately a melancholy story that ever since surgery began the most of the mischief was done by the surgeon himself. It was the willing and tender, though unclean, hand that carried the poison into the wounds." It has been my lot to see several such cases ending fatally, even in my own friends. In the hands of trained gynæcological surgeons it has become the most successful major operation known to surgery, and although removing part of the uterus in some cases with the tumour, yet these patients are able to live perfectly happy married lives, and even spinsters can get married when it was impossible before, on account of the obstruction caused by the fibroid tumour.

Finally, the researches of Lister and Pasteur have assisted gynæcology in the treatment of the fatal disease of carcinoma of the uterus. The only successful treatment at present is abdominal pan-hysterectomy. The immediate risks are great, but the remote consequences are much better than removal by the vaginal route. It only must be remembered that the peritoneum must on no account be fouled by the cancer material, or secondary growth is certain. In the last two years I have been lucky enough to do seven cases successfully, without any return at the present time.

I must ask your forgiveness if in my keenness and interest in a subject to which I have devoted so much study and practice I have tried your kind attention severely. It is a difficult subject and not the most pleasant, yet it cannot be overlooked that it affects not only the present generation, but even more the generations of the future, and is connected directly or indirectly with every branch of medical and surgical work.

